# Application of Immunoprofiling Using Multiplexed Immunofluorescence Staining Identifies the Prognosis of Patients with High-Grade Serous Ovarian Cancer

**DOI:** 10.3390/ijms22179638

**Published:** 2021-09-06

**Authors:** Shin-Wha Lee, Ha-Young Lee, Sung Wan Kang, Min Je Kim, Young-Jae Lee, Chang Ohk Sung, Yong-Man Kim

**Affiliations:** 1 Department of Obstetrics and Gynecology, Asan Medical Center, University of Ulsan College of Medicine, Seoul 05505, Korea; ymkim@amc.seoul.kr; 2 Asan Institute for Life Science, Seoul 05505, Korea; leehayoung@gmail.com (H.-Y.L.); iamksw@naver.com (S.W.K.); kimminje1007@gmail.com (M.J.K.); 3 Department of Obstetrics and Gynecology, GangNeung Asan Hospital, University of Ulsan College of Medicine, Gangneung 25440, Korea; lyjobgy@amc.seoul.kr; 4 Department of Pathology, Asan Medical Center, University of Ulsan College of Medicine, Seoul 05505, Korea; co.sung@amc.seoul.kr

**Keywords:** ovarian cancer, immunoprofiling, CD8, PD-L1, FoxP3, multiplex IHC, quantitative analysis

## Abstract

Immunoprofiling has an established impact on the prognosis of several cancers; however, its role and definition in high-grade serous ovarian cancer (HGSOC) are mostly unknown. This study is to investigate immunoprofiling which could be a prognostic factor in HGSOC. We produced tumor microarrays of 187 patients diagnosed with HGSOC. We performed a multiplexed immunofluorescence staining using Opal Multiplex IHC kit and quantitative analysis with Vectra-Inform system. The expression intensities of programmed death-ligand 1 (PD-L1), CD4, CD8, CD20, FoxP3, and CK in whole tumor tissues were evaluated. The enrolled patients showed general characteristics, mostly FIGO stage III/IV and responsive to chemotherapy. Each immune marker showed diverse positive densities, and each tumor sample represented its immune characteristics as an inflamed tumor or noninflamed tumor. No marker was associated with survival as a single one. Interestingly, high ratios of CD8 to FoxP3 and CD8 to PD-L1 were related to the favorable overall survival (77 vs. 39 months, 84 vs. 47 months, respectively), and CD8 to PD-L1 ratio was also a significant prognostic factor (HR 0.621, 95% CI 0.420–0.917, *p* = 0.017) along with well-known clinical prognostic factors. Additionally, CD8 to PD-L1 ratio was found to be higher in the chemosensitive group (*p* = 0.034). In conclusion, the relative expression levels of CD8, FoxP3, and PD-L1 were significantly related to the clinical outcome of patients with HGSOC, which could be a kind of significant immunoprofiling of ovarian cancer patients to apply for treatment.

## 1. Introduction

Epithelial ovarian cancer (EOC) is the most fatal gynecologic cancer, which can be primarily attributed to acquired resistance to chemotherapy and late-stage diagnosis. According to the GLOBOCAN 2018 report, about 300,000 women have been newly diagnosed with ovarian cancer, resulting in more than 180,000 deaths worldwide in 2018 [1]. High-grade serous ovarian cancer (HGSOC), a major subtype of EOC, represents an unfavorable prognosis of less than 40% 5-year survival rate. More than 70% of patients relapse after frontline treatment with debulking surgery and platinum-based chemotherapy because of eventually developed resistance [2]. Since the mid-2010s, a few new drugs have been introduced to the pool of approved chemotherapeutic agents, such as bevacizumab and olaparib [3,4]. To our disappointment, their benefit for overall survival is minimal at best in patients with relapsed EOC, so the worst prognosis of EOC among gynecologic cancers has not improved significantly over the decades [5,6]. A new, effective treatment strategy is urgently needed to overcome drug resistance while simultaneously preventing metastasis and cancer progression from reducing the survival of patients with EOC.

Immune checkpoint inhibitors (ICIs) are revolutionizing the treatment of cancers and have demonstrated their promising efficacy against various malignancies. In particular, several programmed cell death l (PD1) and programmed death-ligand 1 (PD-L1) blockade therapies have been approved by FDA [7]. In an initial study of EOC [8], nivolumab, an anti-PD1 antibody, showed 15% overall response in platinum-resistant relapsed EOC, including two patients showing durable complete response. However, the clinical benefit of PD1/PD-L1 blockade is relatively not good in EOC compared with melanoma or non-small-cell lung cancer (NSCLC). Moreover, considering that many recent studies have reported that only a few patients respond to immunotherapy, it seems more important to accurately predict the prognosis of patients with EOC and make appropriate treatment plans accordingly.

Pre-existing antitumor immunity has become a significant prognostic factor in diverse cancers, although many previously proven clinical factors are still important [9,10]. Three tumor immune phenotypes described as inflamed, excluded infiltrate, and immune desert were proven to be related to the response to immunotherapy and overall survival (OS) [10]. According to the tumor immune status based on TIL infiltration, inflamed cancer is defined as the presence of a high density of CD8+ T cells in the tumor bed, and it could be well controlled by immunotherapies acting on T cell checkpoint involved in immune tolerance. In contrast to the inflamed cancer, the immune desert tumor is characterized by the absence of T cells in tumor beds; as a result, immunotherapies for T cell priming are bound to fail [11]. EOC is classified as one of the human cancers for which tumor-infiltrating lymphocytes (TILs) were associated with high treatment response and favorable survival [12]. In a review, cytotoxic T lymphocytes (CTLs) and memory T cells are correlated with a good prognosis in many types of cancer, and, interestingly, EOC shows higher correlations with immune cell infiltrates compared with 20 different cancer types [13]. However, evaluating the immune activity in EOC and applying it to the clinical era for patient classification or to decide a treatment plan has not been established. Despite the existence of research that demonstrates the importance of PD-L1 and CD8 expression in tumor cells or TILs as prognostic markers in patients with HGSOC [14,15,16,17,18,19], until now, limited data have been reported, and they have revealed inconsistent results.

We investigated the expression level of several representative immune markers using a multiplex immunohistochemistry (IHC) assay and quantitative analysis in this study. We further demonstrated the association between the expression ratio of immune markers and response to chemotherapy and survival in patients with HGSOC, suggesting that immunoprofiling would be a potent prognostic factor in ovarian cancer.

## 2. Results

### 2.1. Quantitative Analysis Reveals Immune Cellular Densities Are Diverse 

**Figure 1:**
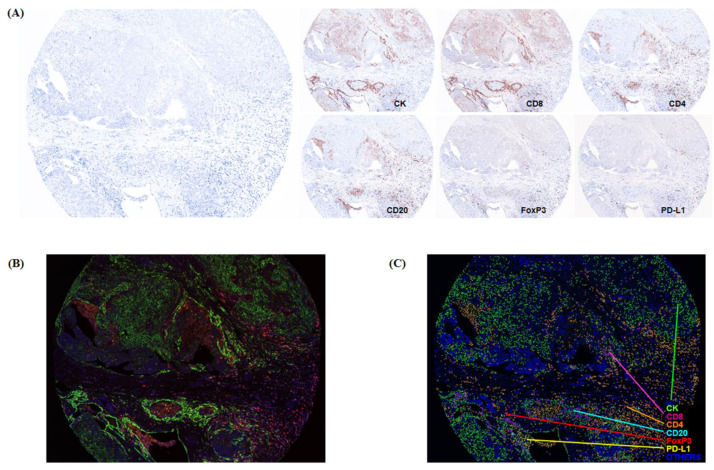
The Opal multiplexed immunohistochemical staining and quantitative analysis. (**A**) The simultaneous staining result of multiple biomarkers, including CK, PD-L1, CD20, CD4, CD8, and FoxP3. (**B**) A composite image was produced according to the spectral library for each fluorescent probe (Opal 520, Opal 540, Opal 570, Opal 620, Opal 650, Opal 690, and DAPI). (**C**) Automatic phenotyping of HGSOC TMA tissue as a previously designated color (green, CK; yellow, PD-L1; sky blue, CD20; orange, CD4; pink, CD8; red, FoxP3; blue, others).

**Figure 2:**
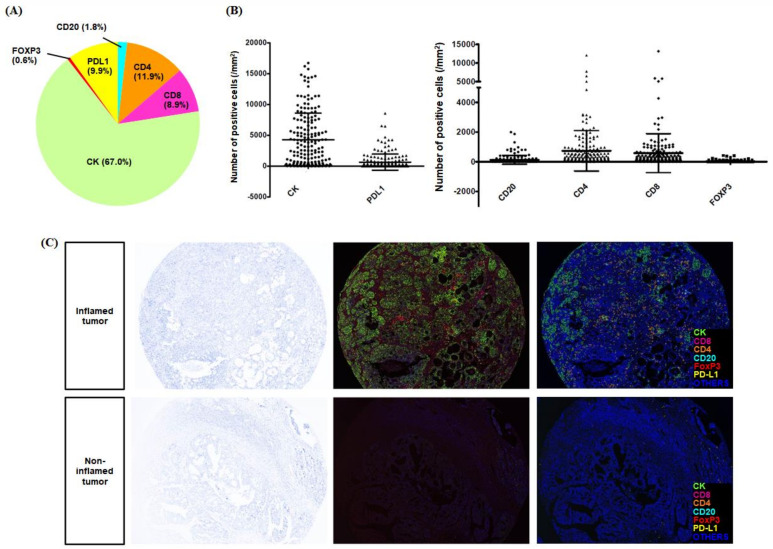
The diverse immune cellular densities and the quantitative values of each immune marker. We obtained the simultaneous staining result of multiple biomarkers, including CK, CD8, CD4, CD20, FoxP3, and PD-L1. After simultaneous staining, automated phenotyping was possible, and the quantitative analysis for positive cellular densities of positive cells was conducted. (**A**) Among six markers, the positive proportion was high in orders of CD4, PD-L1, CD8, CD20, and FoxP3, excluding CK. (**B**) The quantitative value of each marker was shown individually different. (**C**) The inflamed tumors were clearly indicated in the phenotyping view of Opal staining, as shown in the middle view.

Figure 1 shows the typical images of multispectral IHC staining and the process of the multiplexed immunofluorescence imaging system. Each cell detected by DAPI was categorized as specific phenotypes according to the expression of immune markers, and the decision of phenotyping was trained with high confidence (≥60%). Each immune marker showed diverse positive densities, as expressed in Figure 2. Among five immune biomarkers, in terms of the proportion of each type of immune infiltrates, the positivity of CD4 was the highest. The immunosuppressive markers, such as PD-L1 and FoxP3, were shown in 9.9% and 0.6%, respectively (Figure 2A). The CK was expressed in all cancer tissues, including those with considerable variation, and other immune markers showed diverse cellular densities individually (Figure 2B). In the Opal multiplex IHC assay, we could easily discriminate the tumor characteristics of the inflamed and noninflamed tumors because the difference in cellular densities was remarkable, especially in automated phenotyping views (Figure 2C).

### 2.2. A Single Marker Alone Does Not Affect the Survival Rate

**Figure 3:**
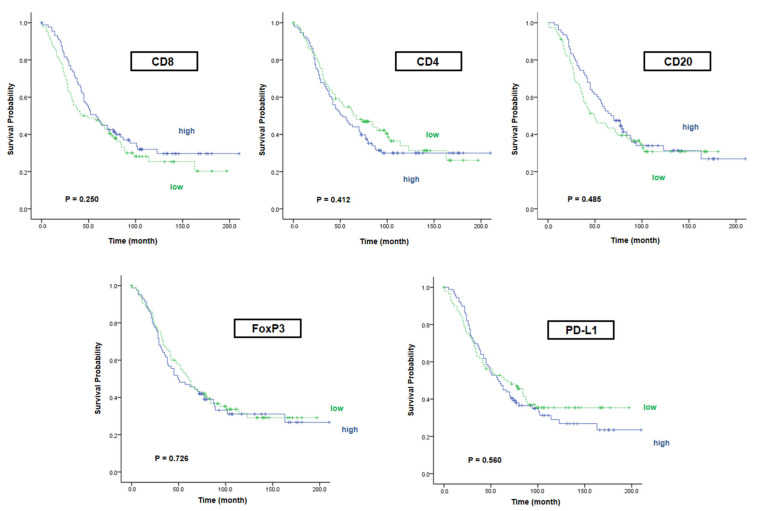
Overall survival according to each immune marker. We classified patients according to the degree of marker expression and analyzed the overall survival according to each marker. As a result, any single immune marker was not related to survival rate.

We divided 187 patients with HGSOC into higher expressed group and lower expressed group based on the median value of each marker except CK. The median OS in the high CD8/low CD8 group was 58 and 42 months, respectively (*p* = 0.250). In the case of CD4 and CD20, there was no significant difference in survival rates either (median OS in CD4: 52 vs. 67 months, *p* = 0.412) (median OS in CD20: 67 vs. 50 months, *p* = 0.485). In addition, the median OS in the high FoxP3 group was not different compared with the low FoxP3 group (50 vs. 61 months, *p* = 0.726). Likewise, the difference between survival rates of patients with high PD-L1 and low PD-L1 was not significant (58 vs. 67 months, *p* = 0.560). As a result, any single immune marker was not related to survival rate, including CD8, which was a representative marker of CTL; FoxP3 in Treg; and PD-L1, which has been known as a prognostic marker in ovarian cancer but is under debate (Figure 3).

### 2.3. The Ratios of CD8 vs. FoxP3/PD-L1 Are Associated with the Survival

**Figure 4:**
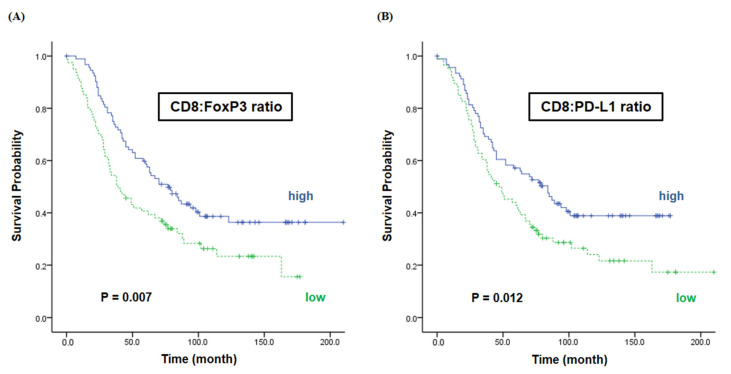
Overall survival according to the ratios of CD8 vs. FoxP3/PD-L1. We classified patients according to the ratio of marker expression, CD8 to FoxP3 and CD8 to PD-L1, and analyzed overall survival according to their ratio. As a result, these two expression ratios of immune markers were significantly associated with survival rate. (**A**) The median OS was 77 months in the high CD8 to FoxP3 ratio group and 39 months in the low CD8 to FoxP3 ratio group (*p* = 0.007). (**B**) The median OS was 84 months in the high CD8 to PD-L1 ratio group and 47 months in the low CD8 to PD-L1 ratio group (*p* = 0.012).

Interestingly, we found that the expression of immune markers with different characteristics in the tumor microenvironment affected the survival rate when measured in their proportion. The survival in the high ratio of CD8 to FoxP3 group was better than in the low ratio group (77 vs. 39 months, *p* = 0.007) (Figure 4A). Furthermore, the median OS was significantly different between the high and low CD8 to PD-L1 ratio groups, with 84 months and 47 months, respectively (*p* = 0.012) (Figure 4B).

### 2.4. CD8:PD-L1 Ratio Is a Predictive and Prognostic Biomarker in HGSOC

**Figure 5:**
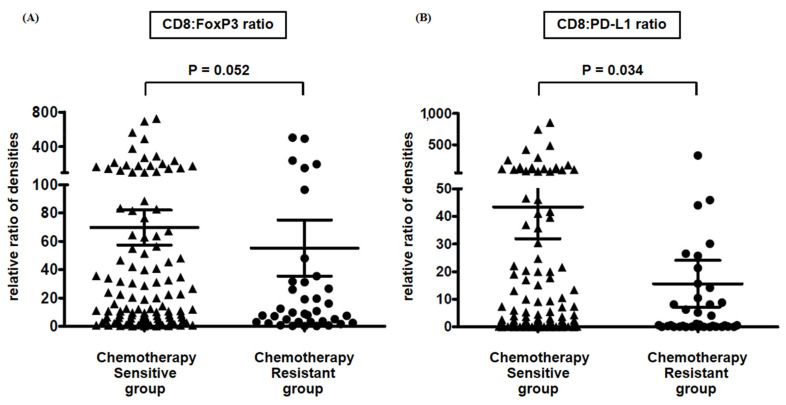
Platinum sensitivity according to ratios of CD8:FoxP3 and CD8:PD-L1. We classified patients according to their clinical response to frontline chemotherapy (paclitaxel/carboplatin) and analyzed CD8 to FoxP3 or PD-L1 expression ratio in two patient groups. (**A**) CD8:FoxP3 ratio seemed to be high in the chemotherapy-sensitive group; however, it was not significant (*p* = 0.052). (**B**) CD8:PD-L1 ratio was significantly higher in the chemotherapy-sensitive group (*p* = 0.034). (∆, CD8:FoxP3 ratio and CD8:PD-L1 ratio in chemotherapy sensitive group, respectively; ●, CD8:FoxP3 ratio and CD8:PD-L1 ratio in chemotherapy resistant group, respectively).

CD8:PD-L1 ratio is correlated with the chemosensitivity and good prognosis in patients with HGSOC. According to the response to adjuvant chemotherapy based on paclitaxel and carboplatin, 133 patients (71.1%) showed chemosensitivity, and 43 patients (23.0%) showed chemoresistance. When we analyzed the difference between the CD8 to FoxP3 or PD-L1 expression ratio in two patient groups, the CD8:PD-L1 ratio was significantly higher in the chemotherapy-sensitive group (*p* = 0.034) (Figure 5B). Otherwise, the CD8:FoxP3 ratio was not different, although it appeared high in the chemotherapy-sensitive group (*p* = 0.052) (Figure 5A). Additionally, in multivariate analysis of prognostic factors associated with overall survival, platinum resistance was indicated as the most unfavorable factor (HR 4.257, 95% CI: 2.753–6.582, *p* < 0.001), and FIGO stage was another unfavorable factor (HR 1.784, 95% CI: 1.295–2.457, *p* < 0.001) (Table 1). Interestingly, although other clinical parameters were not associated with overall survival, CD8 to PD-L1 ratio was a significant biomarker representing favorable prognosis (HR 0.621, 95% CI: 0.420–0.917, *p* = 0.017).

## 3. Discussion

We evaluated the expression of immune cells, which has been proven important in cancer immunology of EOC, in the present study using multiplexed immunofluorescence and multispectral quantitative image analysis. It was revealed that relative positive ratios of CD8+ and PD-L1+ are significantly associated with survival and platinum sensitivity. Moreover, those of CD8+ and FoxP3+ are related to survival, even though they are not significantly related to platinum sensitivity. These results are significant because, to the best of our knowledge, this is the first study showing meaningful immunoprofiling in HGSOC using novel multiplexed immunofluorescence and quantitatively measuring the intensity of each fluorescent target in whole cancer tissues, not discriminating tumor and tumor-associated cells.

Until now, unfortunately, clinically applicable immunoprofiling has not been proven in ovarian cancer. In colorectal cancer, immune parameters designated “Immunoscore”, a scoring system to summarize the density of CD3+ and CD8+ T cells within tumors and the invasive margin, have been confirmed to be the strongest prognostic factor [20,21,22], indicating that assessment of the immune microenvironment could define a new cancer classification based on differences in prognosis. There are two reports referring to the Immunoscore in HGSOC, which emphasize specific immune properties as strong prognostic factors or indicators of chemosensitivity [23,24]. Bösmüller et al. indicated that combined Immunoscore of CD103 and CD3 of TIL examined by the conventional IHC method is correlated with the prognosis in HGSOC. Hao et al. demonstrated that the immune score, produced from genomic analysis with 16 public cohort datasets, based on IFNγ-inducible chemokines is an independent prognostic signature. Otherwise, most results reported have shown that PD-L1 and CD8 expression levels alone or in combination are significant prognostic markers in HGSOC. Hamanishi et al. showed that the PD-L1-expressing tumor cells and CD8+ T lymphocytes are independent prognostic factors and that they are counter-correlated [15]. These results show many similarities with our research results in terms of good prognosis with high CD8 to PD-L1 expression ratios; of course, there are many differences in the research design, including patient number, the proportion of HGSOC, immunoassay, and detective analysis.

The significance of PD-L1 as a prognostic factor in EOC has been primarily reported in line with our study; however, that has yet been controversial. When looking into previous studies, PD-L1+ lymphocytes, not tumor cells, were reported to have prognostic significance [16]; on the other hand, PD-L1 in tumor cells was important in prognosis, but PD-L1 in TIL was not [17]. Nevertheless, some studies emphasized the influence of PD-L1 expression on stromal TILs in terms of survival [18,19]. A meta-analysis has revealed that PD-L1 may not be a prognostic factor for ovarian cancer [25]. These inconsistent reports concerning the immune property in HGSOC, especially regarding PD-L1, may have resulted from the different testing methods, unquantitative scoring systems used to evaluate markers’ expression, variations in sample size, and spatial tissue differences defined by epithelial region and stroma.

We analyzed the expression of immune markers without distinction between tumor cells and tumor-associated cells to notice the immunoprofiling pattern to be utilized in the clinical setting because the possible explanation for the controversy in immunoprofiling for EOC can be found in the intratumoral complexity of ovarian cancer. Cancer cells and stromal cells are more randomly mixed specifically in HGSOC than other cancer types, and it is difficult to discriminate the invasive margin of cancer. Primary cancer cells derived from ovarian/tubal epithelium or extraovarian sites invade the underlying stroma or spread into the peritoneal cavity, developing multicellular aggregates (MCAs) that cause adhesions and implant on mesothelial cells of the peritoneum [26]. We could not explain their vast interactions, despite separating both tumor and tumor-associated stromal cells. We have therefore endeavored to show the clinical significance of immunoprofiling in the tumor sample. In other words, although the detailed description of immunologic functions depending on the specific territories is significant in diverse tumor types, it is so complicated that it might lead to different interpretations while dealing with EOC samples. Many studies have reported PD-L1 in ovarian cancer so far, nevertheless, we cannot use these methods to evaluate EOC patients. Fortunately, significant homogeneity in TILs at different tumor sites of EOC was found in a high-throughput sequencing study [27]. Their findings enable our study adequately to carry out a tumor biopsy from a single site to investigate TIL activity associated with prognosis or chemosensitivity.

We described that high ratios of CD8 to FoxP3 and CD8 to PD-L1 are significantly related to the overall survival, which is roughly twice as long (77 vs. 39 months, 84 vs. 47 months, respectively), and that CD8 to PD-L1 ratio is also a significant favorable prognostic factor (HR 0.621, 95% CI: 0.420–0.917, *p* = 0.017) along with other clinical factors. Additionally, CD8 to PD-L1 ratio was found to be significantly higher in the chemosensitive group (*p* = 0.034). A great variation in FoxP3 expression seems to result in decreasing importance of the ratio of CD8 to FoxP3 as a biomarker for prognosis and chemosensitivity. Many studies have shown that CD8+ TILs within the epithelial component of EOC have significant implications for more prolonged overall survival [14,15,16,17]. Although the role of PD-L1 expression is still debated, as mentioned above, low expression of PD-L1 combined with higher numbers of intraepithelial CD8+ TILs has been related to more prolonged survival [15,17], supporting our present results. Our data are more objective and reproducible in that we have seen the ratio of quantitative positive cell numbers, unlike that compared with conventional IHC and scoring systems.

We used a novel quantitative multispectral image analysis via the Opal Multiplex IHC kit and Vectra-Inform system to evaluate tumor cells and tumor microenvironment of HGSOC. We intended to mitigate bias based on the conventional IHC method and minimize human factors during manual pathological review. Although IHC is a powerful diagnostic and research tool, it has limitations in quantitative measurements when eyeballing under microscopy, leading to biased results. There are two major advantages to the experimental methods that were used in this study. The first is that we could detect multiple markers simultaneously on 4 μm thick sections of FFPE samples, and the other is that the double positivity scoring method made us examine the adequate number of positively expressed cells. We analyzed the immune markers with the count of positive cells per mm^2^ in this study and compared the relative expression ratio of specific markers related to prognosis and chemosensitivity. This analyzing method is the first attempt in ovarian cancer; however, several studies reported that it was possible to analyze accurately the immune environment in various tumors through the multispectral imaging system [28,29,30].

Our study has several weaknesses and strengths, as already mentioned. First, we conducted this study with selected immune markers that have already been reported significant, so we could not evaluate the comprehensive influence of other immune markers. Second, we could not avoid including selection bias because the samples were all from a single institute and were collected retrospectively. Third, and most importantly, it is necessary to perform further study to validate our analyzing concept, which has not been implemented yet. It would be better to evaluate whole cells within tumor samples without discrimination of tumors or tumor-associated cells, given the complexity in HGSOC. The present study is still only the beginning of this concept, and researchers should find immunoprofiling that helps treat patients with more samples and integrated analysis from diverse confirmatory experimental methods including mRNA, microRNA, copy number variation, point mutation, and indel.

## 4. Materials and Methods

### 4.1. Study Patients and Characteristics

This study was performed with tissue microarray (TMA) samples, which were produced with cancer tissues from 187 patients with HGSOC. The median age was 51 years old (range, 25–78 years). Patients with FIGO stages III and IV comprised more than 80% of the population. Most patients were treated with primary debulking surgery followed by adjuvant chemotherapy with paclitaxel/carboplatin. Neoadjuvant chemotherapy was performed in 7.5% of patients, and optimal debulking surgery was carried out in 66.8% of patients. The median PFS was 17.0 months, and the median OS was 58.0 months (Table 2).

### 4.2. Tissue Microarray

We reviewed formalin-fixed and paraffin-embedded (FFPE) tumor samples of 187 patients diagnosed with HGSOC who underwent primary surgery between 1998 and 2013. TMA was produced by taking duplicate 0.6 mm cores from selective regions of each FFPE block after confirming more than 70% cancer tissue by a gynecologic pathologist. Clinical characteristics of patients, including age at diagnosis, surgical outcome, FIGO stage, chemotherapy sensitivity, disease recurrence, and death, were reviewed through the patients’ records. The protocol of this study was approved by the Institutional Review Board of Asan Medical Center, Seoul, Korea (No. 2020-0764), and all methods were performed in accordance with relevant guidelines and regulations. We have reported a part of this study as a poster in the 33rd annual meeting of the Society for Immunotherapy of Cancer (SITC 2018) [31].

### 4.3. Multiplexed Immunofluorescence

Multiplexed immunofluorescence staining was performed using PerkinElmer Opal 7-Color Manual IHC kit (Perkin-Elmer, Waltham, MA, USA). TMA tissue samples were acquired as 4 μm thick sections and placed on plus charged slides. After deparaffinization, tissue samples were rehydrated and antigen was collected in citrate buffer (pH 6.0) using microwave treatment. The following five steps of multiplex immunohistochemistry were followed consecutively for each marker: blocking was performed with antibody diluent (ARD1001EA, PerkinElmer, Waltham, MA, USA), followed by incubation with primary antibody for 1 h, detection using Opal Polymer HRP Ms + Rb secondary antibody (ARH1001EA, PerkinElmer, Waltham, MA, USA), and visualization using Opal tyramide signal amplification (TSA) plus agent, after which the section was placed in citrate buffer (Ph 6.0) and heated using microwave treatment. The primary antibodies and corresponding TSA used for each protein were as follows: anti-CK (AE1/AE3, M3515, Dako, CA, USA) and Opal 520 for CK, anti-PD-L1 (E1L3N, #13684, Cell Signaling, Danvers, MA, USA) and Opal 540 for PD-L1, anti-CD20 (L26, ab133616, Leica, Wetzlar, Germany) and Opal 570 for CD20, anti-CD4 (EPR6855, NCL-L-CD20-L26, Abcam, Cambridge, MA, USA) and Opal 620 for CD4, anti-CD8 (4B11, NB100-65729, Novusbio, Centennial, CO, USA) and Opal 650 for CD8, and anti-FoxP3 (236A/E7, ab20034, Abcam, Cambridge, MA, USA) and Opal 690 for FoxP3. The methods have been introduced in detail in previous studies [32,33].

### 4.4. Multispectral Imaging and Quantitative Image Analysis

The Vectra 3.0 Automated Quantitative Pathology Imaging System (PerkinElmer, Waltham, MA, USA) was used to obtain spectral information. The stained slides scanned at 10× (multiplexed IHC and hematoxylin/eosin) were reviewed again to select appropriate sites showing cancer portions, not dominantly normal ovarian tissues, which were then scanned at 20×. The image files created by Vectra were analyzed using Inform 2.2 image analysis software (PerkinElmer, Waltham, MA, USA). All stained sections (CK-Opal 520, PD-L1-Opal 540, CD20-Opal 570, CD4-Opal 620, CD8-Opal 650, FoxP3-Opal 690, and DAPI) were used to set up the spectral library, which suggested a reference of the intensity of each fluorescent target extracted from the multispectral data using linear unmixing. We analyzed the expression of immune markers by the double positivity scoring method, which divided the cells into positive and negative intensity based on the threshold for each antibody, provided by the Inform software. We counted the number of cells representing two immune markers simultaneously by exporting the scores of each marker with a double positivity scoring method. The numbers per mm^2^ of CK, PD-L1, CD20, CD4, CD8, and FoxP3 positive cells were counted in each stained section.

### 4.5. Statistical Analysis

The data were analyzed with the Mann–Whitney U test or chi-square test for expressing the differences in immune cellular densities, and overall survival was investigated using the Kaplan–Meier analysis so that we were able to find significant prognostic immune markers. We excluded causes of death not related to progression of EOC. Additionally, Cox proportional hazard regression was used to find risk factors in overall survival. SPSS 22.0 software (SPSS, Chicago, IL, USA) and GraphPad Prism 5.0 software (GraphPad Software, La Jolla, CA, USA) were used for statistical analyses. All *p*-values < 0.05 were considered to be statistically significant.

## 5. Conclusions

In conclusion, the relative expression levels of CD8, FoxP3, and PD-L1 were significantly related to the clinical outcome of patients with HGSOC, showing that they could be a kind of significant immunoprofiling in ovarian cancer. Our findings suggest that more diverse approaches are needed to understand the immunoprofiling of patients with ovarian cancer for treatment applications.

## Figures and Tables

**Table 1:** Multivariate analysis of prognostic factors associated with overall survival.

		Hazard Ratio	95% CI	*p*-Value
FIGO stage		1.784	1.295–2.457	<0.001
Surgical outcome	Optimal	1		
	Nonoptimal	1.696	0.786–2.284	0.193
Platinum resistance	Sensitive	1		
	Resistant	4.257	2.753–6.582	<0.001
CD8:FoxP3 ratio	Low	1		
	High	0.521	0.221–1.067	0.106
CD8:PD-L1 ratio	Low	1		
	High	0.621	0.420–0.917	0.017

**Table 2:** Clinical and demographic characteristics of the patients.

	Number of Patients
Total number of patients with HGSOC	187
Age (years), median and range	51 (25–78)
FIGO stage	
Stage I	19 (10.2%)
Stage II	10 (5.3%)
Stage III	143 (76.5%)
Stage IV	15 (8.0%)
Neoadjuvant chemotherapy	
Done	14 (7.5%)
Not done	173 (92.5 %)
Surgical outcome	
Optimal (residual < 1 cm) debulking	125 (66.8%)
Nonoptimal debulking	49 (26.2%)
Not reported	13 (7.0%)
Clinical chemotherapy sensitivity	
Sensitive	133 (71.1%)
Resistant	43 (23.0%)
Not done *	6 (3.2%)
Not reported **	5 (2.8%)
F/U duration (months), median and range	46 (0–194)
Clinical outcome	
PFS (months), median	17.0
3-year PFS	35.1%
OS (months), median	58.0
5-year OS	50.0%

* Two patients were not treated with adjuvant chemotherapy because of FIGO stage IA1, and four patients could not receive the chemotherapy because of postoperative complications. ** Five patients were transferred to another hospital for adjuvant chemotherapy after debulking surgery. HGS-OC, high-grade serous ovarian cancer; FIGO, the International Federation of Gynecology and Obstetrics; F/U, follow-up; PFS, progression-free survival; OS, overall survival.

## Data Availability

The raw/processed data required to reproduce these findings cannot be shared at this time as the data also form part of an ongoing study.
